# Analysing the relationship between lncRNA and protein-coding gene and the role of lncRNA as ceRNA in pulmonary fibrosis

**DOI:** 10.1111/jcmm.12243

**Published:** 2014-04-06

**Authors:** Xiaodong Song, Guohong Cao, Lili Jing, Shengcui Lin, Xiaozhi Wang, Jinjin Zhang, Meirong Wang, Weili Liu, Changjun Lv

**Affiliations:** a Medicine Research Center, Binzhou Medical UniversityYantai, China; b Department of Respiratory Medicine, Affiliated Hospital to Binzhou Medical UniversityBinzhou, China; c Department of Pathology, Affiliated Hospital to Binzhou Medical UniversityYantai, China; d Department of Respiratory Medicine, Affiliated Hospital to Binzhou Medical UniversityYantai, China; e Clinical Laboratory, Affiliated Hospital to Binzhou Medical UniversityYantai, China

**Keywords:** pulmonary fibrosis, lncRNA, ceRNA, MRAK088388, MRAK081523

## Abstract

Long non-coding RNAs (lncRNAs) are involved in various pathophysiologic processes and human diseases. However, their dynamics and corresponding functions in pulmonary fibrosis remain poorly understood. In this study, portions of lncRNAs adjacent or homologous to protein-coding genes were determined by searching the UCSC genome bioinformatics database. This was found to be potentially useful for exploring lncRNA functions in disease progression. Previous studies showed that competing endogenous RNA (ceRNA) hypothesis is another method to predict lncRNA function. However, little is known about the function of ceRNA in pulmonary fibrosis. In this study, we selected two differentially expressed lncRNAs MRAK088388 and MRAK081523 to explore their regulatory mechanisms. MRAK088388 and MRAK081523 were analysed as long-intergenic non-coding RNAs (lincRNAs), and identified as orthologues of mouse lncRNAs AK088388 and AK081523, respectively. qRT-PCR and *in situ* hybridization (ISH) showed that they were significantly up-regulated, and located in the cytoplasm of interstitial lung cells. We also showed that MRAK088388 and N4bp2 had the same miRNA response elements (MREs) for miR-200, miR-429, miR-29, and miR-30, whereas MRAK081523 and Plxna4 had the same MREs for miR-218, miR-141, miR-98, and let-7. Moreover, the expression levels of N4bp2 and Plxna4 significantly increased in fibrotic rats, and were highly correlated with those of MRAK088388 and MRAK081523, respectively. Among their shared miRNAs, miR-29b-3p and let-7i-5p decreased in the model group, and were negatively correlated with the expression of MRAK088388 and MRAK081523, respectively. MRAK088388 and MRAK081523 could regulate N4bp2 and Plxna4 expression by sponging miR-29b-3p and let-7i-5p, respectively, and possessed regulatory functions as ceRNAs. Thus, our study may provide insights into the functional interactions of lncRNA, miRNA and mRNA, and lead to new theories for the pathogenesis and treatment of pulmonary fibrosis.

## Introduction

Recent high-throughput studies on mammalian transcriptome have revealed that tens of thousands of sites are transcribed to produce transcripts. However, most of these transcripts are not translated to proteins, and are called non-coding RNA (ncRNA) [1]. Compared with small ncRNAs, such as siRNAs, miRNAs and piRNAs, long ncRNAs (lncRNAs) are a novel class of mRNA-like transcripts with sizes ranging from 200 bp to >100 kb, and are transcribed by RNA polymerase II [2]. Increasing evidence revealed that lncRNAs have key functions in regulating diverse biological processes, such as imprinting control, cell differentiation, development, immune response, cell cycle and apoptosis [3,4]. However, most lncRNAs remain poorly studied. Thus, identifying lncRNAs and predicting their functions are important. Many lncRNAs act as cis-regulators because their expression is significantly correlated with their neighbouring protein-coding genes [5]. For example, an lncRNA is co-expressed with its bilateral coding genes, Fank1 and Adam12, and its down-regulation reduces the expressions of both coding genes by establishing active chromatin structures [6]. Not every gene becomes silenced because of the transcription of nearby lncRNAs. In many cases, lncRNA transcription may positively or negatively affect the expression of nearby genes [7]. Thus, the positional relationship between lncRNA and mRNA in the genome is important for predicting lncRNA regulation of nearby genes. A previous study demonstrated that some lncRNAs can serve as a ‘sponge’ to titrate microRNAs and prevent them from binding to mRNAs based on the competing endogenous RNA (ceRNA) hypothesis [8]. Pandolfi *et al*. reported that endogenous miRNA decoys have important functions in various biological processes and cell types, and ceRNAs can be found in all organisms that use miRNAs to regulate gene expression. Given the prominent functions of ceRNAs in physiology, their deregulation is a common occurrence in various diseases that can promote their progression [9,10]. Salmena *et al*. has been suggested that all types of RNA transcripts (mRNA, pseudogenes, lncRNA, etc.) can crosstalk with each other by competing for miRNAs through shared miRNA-binding sites [‘miRNA response elements’ (MREs)], thereby acting as ceRNA [11]. Cesana *et al*. showed that a long-intergenic ncRNA (lincRNA), linc-MD1, regulates muscle differentiation by interacting with two miRNAs, miR-135 and miR-133, which can bind to MAML1 and MEF2C to regulate their expression levels. Therefore, identifying well-established miRNAs that bind lncRNAs may help to infer the function of lncRNAs.

The mechanism of expression of lncRNAs remains obscure. Some studies showed evidence for regulation by similar mechanisms, including epigenetic mechanisms, as protein-coding genes, such as promoter methylation, histone deacetylation and miRNAs [12–14]. miR-211 promotes cell growth by repressing lncRNA loc285194 expression. The formation of a reciprocal repression feedback loop is similar to the microRNA-mediated silencing of protein-coding genes [15]. The mutations and altered expression of lncRNAs is closely related to diverse human diseases, ranging from neurodegeneration to cancer [16]. However, their function in pulmonary fibrosis has not been expounded. Idiopathic pulmonary fibrosis (IPF) is a chronic, progressive and devastating interstitial lung disease with no known aetiology or definite treatment modality [17]. Despite the significant progress in our understanding of pulmonary fibrosis, including the function of ageing and cellular senescence, apoptosis, oxidative stress, epithelial-mesenchymal transition (EMT), endoplasmic reticulum stress and miRNA [18], the molecular mechanisms of IPF remain unclear.

In our previous work, we described differentially expressed lncRNAs and mRNAs and related signalling pathways between bleomycin-induced pulmonary fibrosis and normal lung tissue, but the lncRNA function was poorly clarified [19]. In the present study, the relationship between lncRNAs and their adjacent or homology protein-coding genes was assessed, and putative miRNA-target sites in lncRNAs were predicted. Based on the results, lncRNA transcription could affect the expression of adjacent genes in cis, hybridize to the overlapping sense transcript, act as ceRNA or act in more complex ways to regulate gene expression in pulmonary fibrosis. The results will provide insights into the functional interactions of lncRNA, miRNA and mRNA, and lead to new theories for the pathogenesis and treatment of pulmonary fibrosis.

## Materials and methods

### Bleomycin-induced pulmonary fibrosis model

Sprague–Dawley (SD) rats of 8- to 12-week were provided by Yantai Green Leaf Experimental Animal Center. Twenty SD rats were randomly divided into two groups (10 rats each), namely, the normal control group and the pulmonary fibrosis model group. The rats in the model group were administered with 5 mg/kg bleomycin (Bleocin from Daiichi Pure Chemicals Co. Ltd., Tokyo, Japan) dissolved in sterile PBS through a single intratracheal instillation under anaesthesia [20]. The normal control rats were administered with an equal volume of saline. The rats were killed, and their lung tissues were harvested 28 days after bleomycin treatment based on our previous studies [21]. All animal experiments were performed in accordance with the regulations set by the Committee on the Ethics of Animal Experiments of Binzhou Medical University.

### Haematoxylin and eosin and Masson's trichrome staining

After fixing with 4% paraformaldehyde overnight, dehydrating in 70% ethanol and clearing with xylene, the lung tissues were embedded in paraffin wax. Lung tissues of 4 μm-thick sections were prepared and stained with haematoxylin and eosin or Masson's trichrome. Nine random areas were examined at a magnification of ×400. The severity of fibrosis was blindly determined by a semi-quantitative assay.

### LncRNA microarray and data analysis

In our previous study, differential expression of lncRNA in bleomycin-induced pulmonary fibrosis was explored by using ArrayStar Inc. (Rockville, MD, USA) rat lncRNA microarray. LncRNA detection was performed according to the manufacturer's instructions. The following general steps were carried out. First, total RNA was isolated from lung tissues, and RNA quantity and integrity were assessed. Second, the RNA was linearly amplified and labelled as Cy3-dCTP for hybridization onto the lncRNA expression microarray slide. The hybridized arrays were washed, fixed and scanned by using the Agilent DNA Microarray Scanner. Agilent Feature Extraction software (version 10.7.3.1) and GeneSpring GX v11.5.1 software packages (Agilent Technologies, Englewood, CO, USA) were used to analyse the acquired array images and process quantile normalization and subsequent data. Finally, differentially expressed lncRNAs and mRNAs were identified through fold-change filtering after the quantile normalization of raw data.

### LncRNA sequence analysis

The annotated, full-length lncRNA sequences obtained from RefSeq database were subjected to a BLAT query in the UCSC genome browser against rat RGSC 5.0/rn5 genome assembly to identify the genomic loci and sequence conservation with known genes. The browser can analyse lncRNA relative to adjacent protein-coding genes on the same chromosome and identify the homology to exons of annotated protein-coding genes.

### miRNA-target prediction of MRAK088388 and MRAK081523

miRNA-target prediction was performed by ArrayStar Inc. The miRanda and TargetScan algorithms were combined. The advantages of the two algorithms were integrated to predict arbitrary mRNA or lncRNA with miRNA while ensuring that site measurement criteria had biological significance and no loci type was missed. By observing the 2D structure diagram, the strength of binding can be estimated.

### Quantitative real-time reverse transcriptase polymerase chain reaction (qRT-PCR) analysis

The total RNA extracted from various samples was twofold serially diluted in nuclease-free water. Two micrograms of total RNA was used for the synthesis of the first-strand cDNA. For evaluation of miRNAs expression, cDNA was synthesized by using a TaqMan MicroRNA Transcription kit. qRT-PCR analysis was performed with the Rotor-Gene 3000 Real-time PCR system with the following reaction profiles: pre-denaturation at 95°C for 30 sec. and PCR amplification for 40 cycles at 95°C for 15 sec. and at 60°C for 25 sec. PCR was followed by a melt curve analysis to determine the reaction specificity. The relative gene expression was calculated by using standard ΔΔCt methods by Rotor-Gene 6 Software (Corbett Research, New South Wales, Australia). Primers used in qRT-PCR were shown in Table1.

**Table 1:** Primers of MRAK088388, MRAK081523, N4bp2, Plxna4, β-actin, and U6

Primer	Sequence
MRAK088388 sense	5′-GCCCTCCTATCTGGTATCTTTGAA-3′
MRAK088388 antisense	5′-GAGCTCTTCTCACCTGGATCATC-3′
MRAK081523 sense	5′-CTGGCATTCTTGTCAGCTTGTT-3′
MRAK081523 antisense	5′-GGCCCAGGTAGGGAGAGATATG-3′
N4bp2 sense	5′-CGGGCTCAGGGAAATCCTTT-3′
N4bp2 antisense	5′-TTGCTCGGTTCTGGTTCCAC-3′
Plxna4 sense	5′-TTGGACCACGCAACACTCTT-3′
Plxna4 antisense	5′-CTGTTGCTCCCACCCCTG-3′
β-actin sense	5′-GGAGATTACTGCCCTGGCTCCTA-3′
β-actin antisense	5′-GACTCATCGTACTCCTGCTTGCTG-3′
U6 sense	5′-GCTTCGGCAGCACATATACTAAAAT-3′
U6 antisense	5′-CGCTTCACGAATTTGCGTGTCAT-3′

### *In situ* hybridization

The fixed lung tissues were dehydrated in ethanol, cleared with xylene, transferred to paraffin, and sectioned into 5 μm sizes. The paraffin sections were treated with TritonX-100 to enhance probe penetration after conventional dewaxing to water. The slides were washed with PBS and fixed again in 4% paraformaldehyde. After washing with PBS and pre-hybridization at 40°C for 4 hrs, the slides were hybridized with digoxin-labelled RNA oligonucleotide probes at 40°C overnight. On the next day, the lung tissue sections were washed with different concentrations of saline sodium citrate at 50°C. After adding a blocking solution made of sheep serum at 37°C for 1 hr, the slides were incubated with anti-digoxigenin-alkaline phosphatase antibody (Roche, Berlin, Germany) at 4°C overnight. Finally, the slides were stained by NBT/BCIP solution (Roche), avoiding light, after washing with Tris-NaCl buffer.

RNA oligonucleotide probe of MRAK088388: GCTGAAGAATAGACTGTAAGCTTTTCAGACGGTGTATCAGAAACAAAATGTTTTTATGTG.

RNA oligonucleotide probe of MRAK081523: GAGCCCAGTTGTAACTTGGTAAAGGACCTTTGTTATAATTAATTGTATACCTGTGTATGT.

### Statistical analysis

All data were expressed as the mean ± SD. Statistical analysis was performed with the spss 17.0 software (IBM Corporation, Armonk, NY, USA) by paired-samples *t*-test. The *P* < 0.05 indicated a statistically significant difference.

## Results

### Model identification of pulmonary fibrosis

We used haematoxylin and eosin and Masson's trichrome staining to identify whether the pulmonary fibrosis model in this work was successfully established (Figure S1). Result showed that the alveolar structure was complete and continuous without obvious abnormality and that the alveolar septum was thinner and contained a very small amount of collagen fibres in the normal group. On the contrary, the model group showed that the alveolar structure was damaged, accompanied by pulmonary septa thickening and that the fibroblast focus formation and collagen fibres increased significantly. These results indicated that we had successfully established a bleomycin-induced pulmonary fibrosis model.

### Differential expression of lncRNAs

To preliminarily explore the biological significance of lncRNAs in pulmonary fibrosis, the lncRNA expression profiles in the lung tissue of rats after bleomycin injection for 28 days were determined through microarray analysis. Differentially expressed lncRNAs were identified by fold-change filtering, and the fold-change threshold was ≥2.0. Based on the results, hundreds of lncRNAs were differentially expressed in the bleomycin-induced lung samples compared with the normal control group. The list only shows the partial results for the differentially expressed lncRNAs (Table S1) in the model *versus* those in the normal control group.

### LncRNA relative to adjacent or homologous protein-coding gene

LncRNAs were transcribed in complex loci, which had sequence similarity relative to protein-coding genes. Through chromosomal localization and BLAST sequence alignment, we analysed lncRNAs and their associated protein-coding genes, which could help reveal the function and mechanisms of lncRNAs in pulmonary fibrosis. According to the positional relationship between lncRNA and the adjacent protein-coding genes in the same chromosome, lncRNAs can be roughly classified as sense_exon_overlap, sense_intron_overlap, antisense_exon_overlap, antisense_intron_overlap, bidirectional and intergenic (Table2). The majority of differentially expressed lncRNAs were related to the exons of protein-coding genes from intergenic regions or within the introns of protein-coding genes with the other more complex types that were temporarily difficult to define. Fifteen lncRNAs showed 90% sequence similarity to the exons of protein-coding genes, a majority of which participated in the transcription, translation and substance metabolism. In all cases, the genomic loci of the paired lncRNA and their homologous protein-coding genes were on different chromosomes (Table3).

**Table 2:** Positional relationship between lncRNA and the adjacent protein-coding genes. By sequence analysis, we obtained the relationship of lncRNA with its nearby coding gene and the coordinate of the coding gene

SeqID	Chr	Strand	Relationship	Associated gene_acc	Associated gene_name	Associated protein_name	Associated gene_strand
BC168907	4	+	sense_exon_overlap	NM_001108640	Ttll3	Tubulin tyrosine ligase-like family, member 3	+
BC101922	1	−	sense_exon_overlap	NM_022702	Taok2	TAO kinase 2	−
MRAK010202	9	+	sense_exon_overlap	NM_001134637	Plin5	perilipin 5	+
AF177677	19	+	sense_exon_overlap	NM_053392	Cdh11	cadherin 11	+
MRAK017969	3	+	sense_intron_overlap	NM_001011964	Mapkap1	mitogen-activated protein kinase associated protein 1	+
MRAK153687	1	−	sense_intron_overlap	NM_001107523	chd2	chromodomain helicase DNA binding protein 2	−
MRAK084899	18	−	sense_intron_overlap	NM_001107378	Zfp608	zinc finger protein 608	−
AY168782	18	+	sense_intron_overlap	NM_019191	Smad2	SMAD family member 2	+
MRAK160296	3	+	antisense_exon_overlap	NM_001134983	RGD1564927	similar to TGFB-induced factor 2	−
MRNR_002862	5	−	antisense_exon_overlap	NM_001100538	Pabpc4	poly(A) binding protein, cytoplasmic 4	+
MRBC006619	4	+	antisense_exon_overlap	NM_001034010	Tril	TLR4 interactor with leucine-rich repeats	−
MRAK152421	5	−	antisense_exon_overlap	NM_001173432	Atp13a2	ATPase type 13A2	+
BC091367	5	+	antisense_intron_overlap	NM_001191765	Eif2c1	eukaryotic translation initiation factor 2C	−
BC088244	1	+	antisense_intron_overlap	NM_001024747	Phf10	PHD finger protein 10	−
XR_007395	3	−	antisense_intron_overlap	NM_001107786	Rin2	Ras and Rab interactor 2	+
BC167069	3	−	bidirection	NM_001107762	Nusap1	nucleolar and spindle-associated protein	+
X95079	7	+	bidirection	NM_013194	Myh9	myosin, heavy chain 9	−
MRAK144357	13	−	intergenic				
MRAK018131	1	−	intergenic				
MRNR_002847	1	−	intergenic				
MRAK088388	14	−	intergenic				
MRAK081523	4	−	intergenic				

SeqID: lncRNA name.

Chr: chromosome no. which lncRNA is transcribed.

Strand: the strand of chromosome which lncRNA is transcribed; ‘+’ is sense strand of chromosome, ‘−’ is antisense strand of chromosome.

Relationship: positional relationship between lncRNA and the adjacent protein-coding genes.

‘sense exon overlap’: the lncRNA's exon is overlapping a coding transcript exon on the same genomic strand;

‘sense intron overlap’: the lncRNA is overlapping the intron of a coding transcript on the same genomic strand;

‘antisense_exon_overlap’: the lncRNA is overlapping a coding transcript exon on the different genomic strand;

‘antisense_intron_overlap’: the lncRNA is overlapping a coding transcript on the different genomic strand without sharing overlapping exons;

‘bidirection’: the lncRNA is oriented head to head to a coding transcript within 1000 bp;

‘intergenic’: there are no coding transcripts within 30 kb of the lncRNA.

Associated gene_acc: the number on NCBI database of mRNA which lncRNA is overlapping with.

Associated gene_name: the name of mRNA which lncRNA is overlapping with.

Associated protein_name: the name of protein which is translated from the mRNA that overlapping with a lncRNA.

Associated gene_strand: the strand of chromosome which mRNA is transcribed; ‘+’ is sense strand of chromosome, ‘−’ is antisense strand of chromosome (*n* = 6, replicates).

**Table 3:** Differentially expressed lncRNAs that showed homology to protein-coding genes. Through the BLAT program of the UCSC genome browser, we discovered homology between lncRNAs and protein-coding genes located in different chromosomes. This table shows the chromosomal location of the lncRNA, homologous protein-coding genes, and the official full names of protein-coding genes. lncRNA ID: the name of lncRNA. Mimickd Gene: the short name of lncRNA's homologous gene. Official Full Name: the official full name of lncRNA's homologous gene. Sequence similarity%: the Sequence similarity% between lncRNA and its homologous gene

lncRNA ID	Mimickd Gene	Official Full Name	Chromosome		Sequence similarity %
			No. of LncRNA Gene	No. of mRNA Gene	
XR_009210	Nlk	Nemo like kinase	2	10	99.9
XR_007177	Gzmc	Granzyme C	15	15	96.2
XR_008194	Ube2c	Ubiquitin-conjugating enzyme E2C	12	3	95.7
XR_006792	Rpl21	Ribosomal protein L21	17	12	95.0
XR_005917	Rpl24	Ribosomal protein L24	15	11	93.7
XR_007003	Fabp5	Fatty acid binding protein5, epidermal	Un	2	93.2
XR_009442	Rpl29	Ribosomal protein L29	7	8	93.2
XR_006878	Idh3B	Isocitrate dehydrogenase 3 (NAD+) beta (Idh3B), nuclear gene encoding mitochondrial protein	7	3	91.6
XR_007206	Rps7	Ribosomal protein S7	8	6	91.5
XR_005734	Sdad1	SDA1 domain containing 1	18	14	91.3
XR_007949	Rpl27	Ribosomal protein L27	1	10	91.1
XR_005499	Fabp5	Fatty acid binding protein5, epidermal	3	2	90.9
XR_006774	Rpl18	Ribosomal protein L18	12	1	90.9
XR_007062	Rpl18a	Ribosomal protein L18 A	1	16	90.1
XR_007017	Rpl6	Ribosomal protein L6	3	12	90.0

### miRNA-target sites in MRAK088388 and MRAK081523

**Figure 1:**
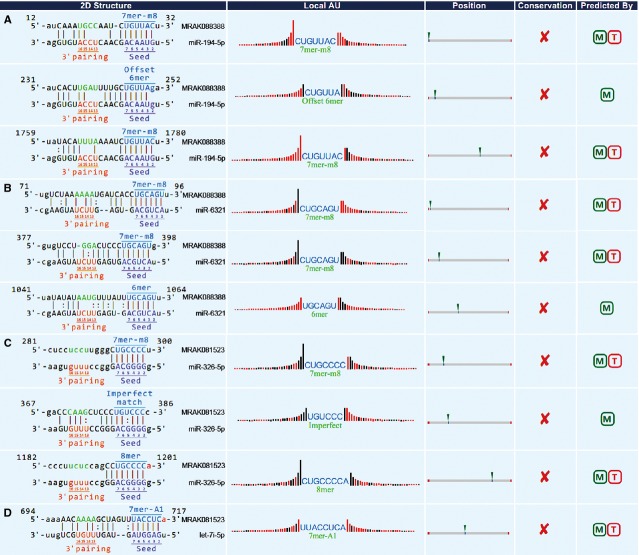
The 2D structure of the miRNA-binding sites on lncRNAs. 2D structure: the specific location of the binding sites on the full-length sequence of lncRNA and the types of binding sites (8mer, 7mer-m8, 7mer-A1, 6mer, offset 6mer, imperfect), as well as the base pairing. ‘|’ Indicates an exact match; ‘:’ indicates G: U pairing. The base pairings at 2–7 and 13–16 are particularly important for site recognition, highlighted in brown. Local AU: weighting of the AU. This feature affects accessibility of binding sites. The red bar indicates that the location is A: U. The redder the bar is, the higher the weighting of the AU. Position: relative position of binding sites on lncRNA. The location closer to the sides is better. Conservation: relatively conservative estimates of seed complementary region and UTR between species. A higher weighting occurs when the conservative seed region is located on the non-conservative UTR. Currently, data for constructing the phylogenetic tree of different species for lncRNAs are not sufficient. The ‘Conservation’ section is meaningless for lncRNA. All sites on lncRNA are treated as non-conserved ones. Predicted By: whether this locus is in accordance with the threshold criterion of prediction algorithms (miRanda, TargetScan). (A) The 2D structure of miR-194-5p on MRAK088388. (B) The 2D structure of miR-6321 on MRAK088388. (C) The 2D structure of miR-326-5p on MRAK081523. (D) The 2D structure of let-7i-5p on MRAK081523.

Through bioinformatics analysis, 227 miRNAs were found to bind to MRAK088388, whereas 132 were found to bind to MRAK081523. Given that lncRNAs do not contain a untranslated regions, miRNA binding–site prediction in lncRNAs was based on their full-length sequence. All sites on lncRNAs were treated as non-conserved sequences. The following six types of seed-matched sites (Fig.1) were found: the 8mer site, which perfectly matches positions 2 to 8 of the mature miRNA (the seed + position 8) followed by an ‘A’; the 7mer-m8 site, which perfectly matches positions 2 to 8 of the mature miRNA (the seed + position 8); the 7mer-A1 site, which perfectly matches positions 2 to 7 of the mature miRNA (the seed) followed by an ‘A’; the 6mer site, which perfectly matches the 6-nt miRNA seed (miRNA nucleotides 2 to 7); the offset 6mer site, which perfectly matches positions 3 to 8 of the miRNA; and the imperfect site, wherein positions 2 to 7 of the miRNA have G:U non-standard pairing or mismatch or are missing. The hierarchy of site efficacy was as follows: 8mer>7mer-m8>7mer-A1>6mer>offset 6mer>imperfect. Based on various scoring results, higher scores were assigned to miRNAs that bound to MRAK088388 (Table4) and MRAK081523 (Table5).

**Table 4:** Predicted miRNAs bind to MRAK088388

Identity	Accession	Site no.	Type of site	Context+	Context	Structure	Energy	Is experimental validated
rno-miR-344i	MIMAT0025049	2	8mer	−0.42	−0.334	297	−32.2	TURE
rno-miR-6316	MIMAT0025053	2	8mer, 7mer-m8	−0.41	−0.611	308	−29.59	TRUE
rno-miR-21-3p	MIMAT0004711	2	8mer, 7mer-m8	−0.408	−0.581	289	−25.18	TRUE
rno-miR-3120	MIMAT0017900	2	7mer-m8	−0.402	−0.536	289	−24.4	TRUE
rno-miR-194-5p	MIMAT0000869	3	7mer-m8 offset 6mer	−0.381	−0.593	442	−41.91	TRUE
rno-miR-126a-3p	MIMAT0000832	1	8mer	−0.358	−0.248	148	−18.86	TRUE
rno-miR-27a-3p	MIMAT0000799	3	7mer-m8	−0.357	−0.708	447	−41.04	TRUE
rno-miR-26b-5p	MIMAT0000797	3	7mer-m8 offset 6mer	−0.348	−0.581	444	−30.64	TRUE
rno-miR-3557-3p	MIMAT0017820	4	8mer 7mer-m8 imperfect	−0.346	−0.503	582	−81.21	TRUE
rno-miR-27b-3p	MIMAT0000798	4	7mer-m8 offset 6mer	−0.334	−0.705	588	−55.75	TRUE
rno-miR-3569	MIMAT0017849	2	8mer offset 6mer	−0.333	−0.305	300	−42.48	TRUE
rno-miR-3573-3p	MIMAT0017857	2	8mer	−0.328	−0.577	298	−30.59	TRUE
rno-miR-449a-5p	MIMAT0001543	1	7mer-m8	−0.327	−0.338	170	−23.12	TRUE
rno-miR-26a-5p	MIMAT0000796	2	7mer-m8	−0.32	−0.507	303	−22.01	TRUE
rno-miR-6324	MIMAT0025063	1	8mer	−0.32	−0.324	154	−17.66	TRUE
rno-miR-1199-5p	MIMAT0031125	1	8mer	−0.318	−0.359	148	−17.44	FALSE
rno-miR-6321	MIMAT0025059	3	7mer-m8 6mer	−0.313	−0.384	457	−50.33	TRUE
rno-miR-6332	MIMAT0025073	2	8mer 7mer-m8	−0.309	−0.253	301	−37.68	TRUE
rno-miR-34a-5p	MIMAT0000815	1	7mer-m8	−0.308	−0.307	164	−21.89	TRUE
rno-miR-758-5p	MIMAT0017316	1	7mer-m8	−0.308	−0.298	166	−19.76	TRUE
rno-miR-29b-3p	MIMAT0000801	1	7mer-m8	−0.303	−0.323	157	−14.55	TRUE
rno-miR-200c-3p	MIMAT0000873	1	8mer	−0.207	−0.414	142	−9.58	TRUE
rno-miR-429	MIMAT0001538	1	8mer	−0.176	−0.408	145	−11.68	TRUE
rno-miR-30a-5p	MIMAT0000808	1	offset 6mer	−0.043	−0.1	142	−13.82	TRUE

Identity: the name of the mature miRNA in miRBase v20.

Accession: the mature accession ID used in miRBase v20.

Site No.: the total number of binding sites.

Type of site: the type of the binding sites.

Context+: the sum of the context+ scores used in TargetScan after version 6.0; the more negative, the better.

Context: the sum of the context scores used in TargetScan before version 5.x; the more negative the value, the better.

Structure: the sum of the structure scores used in miRanda; the higher the score, the better.

Energy: the sum of the free energy as predicted by miRanda; the more negative the value, the better.

Is Experimental Validated: whether this miRNA is validated by experimental data.

**Table 5:** Predicted miRNAs bind to MRAK081523

Identity	Accession	Site no.	Type of site	Context+	Context	Structure	Energy	Is experimental validated
rno-miR-326-5p	MIMAT0017028	3	8mer 7mer-m8 imperfect	−0.442	−0.242	431	−65.97	TRUE
rno-miR-485-5p	MIMAT0003203	2	7mer-m8	−0.343	−0.372	290	−34.96	TRUE
rno-miR-300-5p	MIMAT0004743	1	8mer	−0.338	−0.421	156	−15.16	TRUE
rno-miR-702-5p	MIMAT0017884	1	8mer	−0.317	−0.274	142	−13.86	TRUE
rno-miR-203b-3p	MIMAT0017800	2	7mer-m8	−0.298	−0.421	295	−29.93	TRUE
rno-miR-33-3p	MIMAT0017104	2	8mer 7mer-m8	−0.297	−0.813	305	−22.7	TRUE
rno-miR-466b-3p	MIMAT0017285	1	8mer	−0.295	−0.47	159	−15.26	TRUE
rno-miR-532-5p	MIMAT0005322	1	7mer-m8	−0.268	−0.302	151	−10.71	TRUE
rno-miR-511-5p	MIMAT0012829	1	7mer-m8	−0.268	−0.302	152	−10.37	TRUE
rno-miR-343	MIMAT0000591	1	7mer-m8	−0.262	−0.24	140	−13.75	TRUE
rno-miR-203a-3p	MIMAT0000876	1	8mer	−0.245	−0.47	155	−14.24	TRUE
rno-miR-883-3p	MIMAT0005290	1	7mer-m8	−0.235	−0.268	152	−10.33	TRUE
rno-miR-196c-3p	MIMAT0017299	1	7mer-m8	−0.233	−0.332	162	−14.74	TRUE
rno-miR-764-3p	MIMAT0017370	1	7mer-m8	−0.229	−0.203	143	−16.25	TRUE
rno-miR-122-5p	MIMAT0000827	2	7mer-m8	−0.229	−0.282	292	−32.61	TRUE
rno-miR-27b-5p	MIMAT0017101	1	8mer	−0.226	−0.236	158	−16.17	TRUE
rno-miR-3552	MIMAT0017813	2	7mer-m8 offset 6mer	−0.205	−0.175	294	−46.54	TRUE
rno-miR-3562	MIMAT0017832	1	7mer-m8	−0.196	−0.168	162	−28.28	TRUE
rno-miR-883-5p	MIMAT0017294	1	7mer-m8	−0.194	−0.213	153	−15.92	TRUE
rno-miR-98-3p	MIMAT0017111	1	7mer-m8	−0.121	−0.271	151	−4.22	TRUE
rno-let-7i-5p	MIMAT0000779	1	7mer-A1	−0.115	−0.144	146	−18.16	TRUE
rno-miR-29b-5p	MIMAT0004717	1	7mer-m8	−0.113	−0.23	161	−20.19	TRUE
rno-miR-218a-5p	MIMAT0000888	1	7mer-m8	−0.105	−0.156	162	−17.51	TRUE
rno-miR-141-3p	MIMAT0000846	1	offset 6mer	−0.021	−0.022	141	−13.96	TRUE

Identity: the name of the mature miRNA in miRBase v20

Accession: the mature accession id used in miRBase v20

Site No.: the total number of binding sites

Type of site: the type of the binding sites

Context+: the sum of the context+ scores used in TargetScan after version 6.0; the more negative, the better

Context: the sum of the context scores used in TargetScan before version 5.x; the more negative the value, the better

Structure: the sum of the structure scores used in miRanda; the higher the score, the better

Energy: the sum of the free energy as predicted by miRanda; the more negative the value, the better

Is Experimental Validated: whether this miRNA is validated by experimental data.

**Figure 2:**
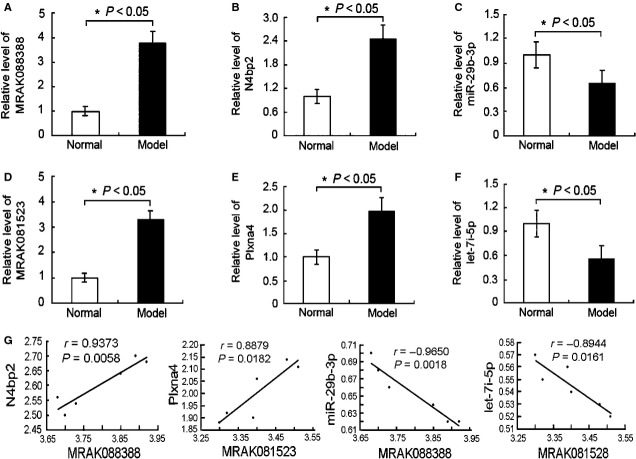
Expression of MRAK088388 and MRAK081523 as well as related protein-coding genes and miRNAs by qRT-PCR. (A) The expression of MRAK088388 was up-regulated in the model group compared with that in the normal group. (B) The expression of N4bp2 was increased in the model group compared with that in the normal group. (C) The expression of miR-29b-3p was reduced in the model group compared with that in the normal group. (D) The expression of MRAK081523 was up-regulated in the model group compared with that in the normal group. (E) The expression of Plxna4 was increased in the model group compared with that in the normal group. (F) The expression of let-7i-5p was decreased in the model group compared with that in the normal group. (G) MRAK088388 and MRAK081523 levels were positive correlation with their respective related protein-coding genes N4bp2 and Plxna4, however, inversely correlated with their respective shared miRNAs miR-29b-3p and let-7i-5p. And statistical analysis was performed by Pearson correlation coefficient respectively; *n* = 6 (rats) with three replicates.

**Figure 3:**
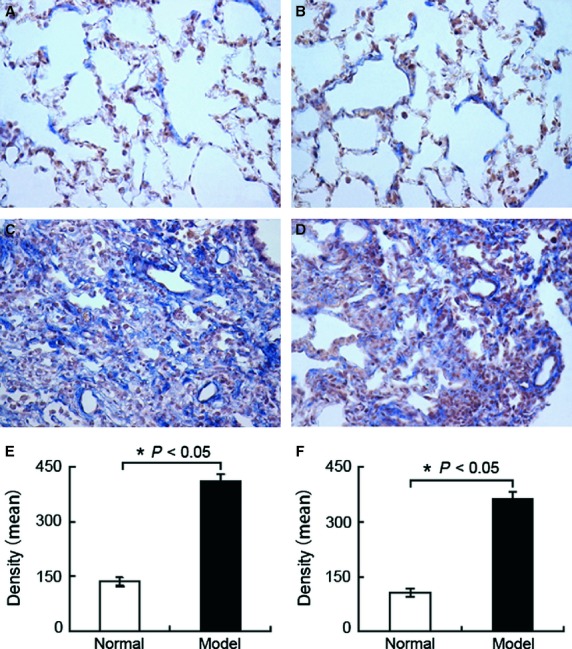
Location and expression of MRAK088388 (A, C and E) and MRAK081523 by *in situ* hybridization (B, D and F). MRAK088388 and MRAK081523 was stained blue in the plasma of interstitial lung cells. (A) Location and expression of MRAK088388 in the normal groups. (B) Location and expression of MRAK081523 in the normal groups. (C) Location and expression of MRAK088388 in the model group. MRAK088388 was up-regulated. (D) Location and expression of MRAK081523 in the model group. MRAK081523 was up-regulated. (E) Density analysis of MRAK088388. (F) Density analysis of MRAK081523. Each bar represents the mean ± SD, *n* = 6. Original magnification, 400×; *P < 0.05.

MRAK088388 and MRAK081523 are involved in ceRNA network MRAK088388 and MRAK081523 were up-regulated (Table S1) and identified from the orthologues of mouse lncRNAs AK088388 and AK081523, respectively, which were obtained from the NONCODE database (http://www.noncode.org). After sequence alignment and chromosomal localization in rats, the two lncRNAs did not overlap with the protein-coding genes, and no protein-coding gene was identified within 30 kb in rats. Therefore, they belonged to lincRNA. Moreover, they had 88.1% and 89.3% sequence similarity to the human protein-coding genes Nedd4 binding protein 2 (N4bp2) and plexin-A4 (Plxna4), respectively. miRNA-target prediction showed that MRAK088388 and N4bp2 had the same MRE for miR-29b-3p, whereas MRAK081523 and Plxna4 had the same MRE for let-7. To identify the ceRNA interaction between MRAK088388 and N4bp2, as well as between MRAK081523 and Plxna4, we detected whether they are co-expressed in lung tissues by using qRT-PCR. The results show that the expression levels of MRAK088388 and MRAK081523 significantly increased in the model group than those in the normal control group, and paralleled with the overexpression of N4bp2 and Plxna4, respectively. By contrast, the levels of their shared miRNAs, miR-29b-3p and let-7i-5p, significantly decreased and was statistically correlated with overexpression lncRNAs respectively (Fig.2). Furthermore, we explored the locations of MRAK088388 and MRAK081523 by ISH, in which a blue–violet colour indicates a positive reaction. MRAK088388 and MRAK081523 were observed in the cytoplasm of interstitial lung cells, which benefited lncRNA function analysis (Fig.3).

## Discussion

By analysing the relationships between lncRNAs and protein-coding genes, as well as by searching putative miRNA-target sites in lncRNAs, we speculate that lncRNAs have important functions in regulating gene expression in pulmonary fibrosis.

LncRNAs can regulate gene expression at the levels of chromatin modification, transcription and post-transcriptional processing [22]. They can regulate the transcriptional process through a range of mechanisms. Initial studies indicated that lncRNAs can regulate neighbouring protein-coding genes through trans- or cis-regulators [23,24]. Most mammalian genes express antisense transcripts, and antisense ncRNAs can mask key cis-elements in mRNA by the formation of RNA duplexes to regulate various steps in the post-transcriptional processing of mRNAs [25]. Given the locations where lncRNAs are transcribed relative to coding regions, lncRNAs can be divided into antisense transcripts of protein-coding genes, bidirectional promoter transcripts, transcripts associated with enhancers or repetitive regions and other transcripts originating from intergenic regions [26]. In our present work, we analysed the positional relationship between lncRNA and protein-coding genes. We have suggested that if the function of protein-coding gene is already known, then lncRNA is predicted to have similar functions. Based on our results, lncRNA AF177677 was an overlapped coding exon of gene CDH11 in sense orientation. Moreover, other studies revealed that CDH11 increases in pulmonary fibrosis, and contributes to EMT [27]. These results may guide us in designing experiments to explore whether AF177677 participates in EMT. Our results show that some lncRNAs did not overlap with any coding gene or had nearby coding genes, defined as lincRNA. Moreover, MRNR_002847 was a lincRNA derived from the sequence alignment with NR_002847, which is a lincRNA in mice called metastasis-associated lung adenocarcinoma transcript 1 (MALAT1). MALAT1 is up-regulated in many solid tumours [28], and is associated with cancer metastasis and recurrence [29,30]. Ying *et al*. found that up-regulated MALAT1 contributes to bladder cancer cell migration by inducing EMT [31], which is also an important manifestation of pulmonary fibrosis. On the basis of these points, we could explore the function of MALAT1 in EMT through the ceRNA language.

Given that our previous assay also contained probes for known protein-coding genes, we could determine whether the lncRNA-related protein-coding genes are simultaneously differentially expressed in bleomycin-induced pulmonary fibrosis. The results show that four lncRNAs (BC168907, BC101922, MRAK010202, and MRAK084899) and their corresponding protein-coding genes (Ttll3, Taok2, Plin5, and Zfp608) were down-regulated. X95079 was up-regulated, and its accompanying related protein-coding gene Myh9 was down-regulated (Table2). This finding was similar to a recent genome-wide analysis of lncRNA expression in uraemia patients reported by Sui *et al*., who discovered the expression changes in lncRNAs and their associated protein-coding genes in the same or opposite direction [32]. Zhu *et al*. confirmed that overexpressing RERT-lncRNA (an lncRNA whose sequence overlaps with Ras-related guanosine triphosphate-binding protein 4b and EGLN2) up-regulates EGLN2 [33]. Thus, we inferred that the differentially expressed lncRNA in bleomycin-induced pulmonary fibrosis may positively or negatively regulate the expression of their neighbouring protein-coding genes. Although the remaining lncRNA-related protein-coding genes exhibited no expression changes, these genes, such as RGD1564927 (similar to TGFB-induced factor 2, SMAD family member 2, and mitogen-activated protein kinase associated protein 1), have already been proven as important factors in pulmonary fibrosis [34–36]. Other protein-coding genes are mainly associated with transcription, translation, energy metabolism and signal transduction (Table2). The abnormality of these biological processes may be involved in the development of pulmonary fibrosis. In summary, the differential expression of lncRNA and its sequence or positional relationship with the protein-coding genes are of great significance in pulmonary fibrosis.

However, lncRNAs may regulate gene expression in many ways during pulmonary fibrosis. New studies showed that lncRNAs act as key ceRNAs [8,37,38], thereby greatly enhancing the functionality of lncRNAs.

The ceRNAs affect the distribution of miRNA on their targets and impose an additional level of post-transcriptional regulation. A recent study reported that the phosphatase and tensin homolog pseudogene (PTENP1) is an lncRNA that sequesters numerous PTEN-targeting miRNAs to regulate PTEN transcription and translation by acting as an miRNA sponge [39]. Moreover, Dharap *et al*. found that 61 stroke-responsive lncRNAs show >90% sequence homology to protein-coding genes, and these lncRNAs may be pseudogenes [40]. Given that these lncRNAs are highly homologous to protein-coding genes, they may have common MREs and influence expression levels by competing for shared miRNAs. Thus, we proposed that some lncRNAs may function as ceRNAs to link the miRNAs and transcriptional network in pulmonary fibrosis.

We subsequently explored whether two differentially expressed lncRNAs MRAK088388 and MRAK081523 act as a natural decoy for miRNAs. In this work, we found that changes in MRAK088388 and MRAK081523 expression were similar to those in N4bp2 and Plxna4 expression, respectively, in fibrotic lung tissues. Given that MRAK088388 and MRAK081523 had high sequence similarity to N4bp2 and Plxna4, we speculated that they may have the same MREs. First, we identified MRAK088388- and MRAK081523-targeting miRNAs. We used TargetScan and miRanda database queries to obtain miRNAs, which had higher targeting combined with N4bp2, namely, miR-200, miR-429, miR-29 and miR-30. These miRNAs also had binding sites on MRAK088388 (Table4). N4bp2 is a Bcl-3 binding protein and Bcl-3 is an oncoprotein. A study showed that the expression of these genes is co-upregulated in tumour tissue [41]. A more recent study found that IPF exhibits several cancer-like pathogenic features; lung myofibroblasts, which are similar to cancer cells, acquire epigenetic and genetic abnormalities as well as functional features, such as uncontrolled proliferation, resistance to apoptosis, and high migration rates [42]. The high expression of N4bp2 in our study may be related to myofibroblast growth. Moreover, our ISH results show that MRAK088388 was expressed mainly in lung interstitial cells. Thus, N4bp2 and MRAK088388 may have the same functions. Among their shared miRNAs, previous research showed that miR-29 is significantly down-regulated and expressed in mesenchymal cells in the lungs of bleomycin-treated mouse [43]. miR-29 was used to investigate the miRNA dependency of ceRNA-mediated MRAK088388 regulation. Our qRT-PCR results show that the expression of miR-29 and MRAK088388 was highly correlated in lung tissue. Based on these results and preliminary analysis, MRAK088388 possibly regulated lung myofibroblast growth and subsequent collagen deposition by sponging miR-29, which could bind to N4bp2.

By the same method, we found that MRAK081523 and Plxna4 had the same MREs for miR-218, miR-141, miR-98 and let-7. Plxna4 reportedly promotes tumour progression and tumour angiogenesis by enhancing VEGF and basic fibroblast growth factor signalling [44]. Moreover, VEGF is an important regulator of angiogenesis that promotes the development of IPF [45]. Thus, the high expression of N4bp2 in our study may be related to angiogenesis. Similar to MRAK088388, MRAK081523 was located in lung interstitial cells. Let-7 isoform let-7d expression significantly decreases and has a key regulatory function in IPF [46], but the function of let-7i has not been reported. Based on our present study, which showed the lower expression of let-7i in lung tissues of bleomycin-treated rat than that in normal rats, we used let-7i to determine if MRAK088388 regulation is ceRNA-mediated miRNA-dependent. Our qRT-PCR results show that the expression of let-7i and MRAK081523 was highly negatively correlated. MRAK081523 may compete with the let-7i pool to regulate the expression of Plxna4 and participate in angiogenesis.

In this study, many lncRNAs were differentially expressed in bleomycin-induced lung fibrotic rats, and their functions could be predicted based on their positional relation with protein-coding genes and ceRNA network. However, this research is still in the exploratory stage, and conclusions were obtained only through the changes in their levels and bioinformatics analysis, which need experimental identification and validation. Our assumption may also provide the first evidence that ceRNA interaction exists in pulmonary fibrosis, and give new potentially therapeutic targets in pulmonary fibrosis. In the future, we should design experiments, such as the gain-of-function and loss-of-function, to investigate thoroughly the ceRNA language between them.

## Supporting Information

**Figure S1** Model identification of pulmonary fibrosis by haematoxylin and eosin and Masson staining.

**Table S1** Differentially expressed lncRNAs screening.

## References

[1] Mattick JS, Makunin IV (2006). Non-coding RNA. Hum Mol Genet.

[2] Caley DP, Pink RC, Trujillano D (2010). Long noncoding RNAs, chromatin, and development. Sci World J.

[3] Nagano T, Fraser P (2011). No-nonsense functions for long noncoding RNAs. Cell.

[4] Lee JT (2012). Epigenetic regulation by long noncoding RNAs. Science.

[5] Paraskevopoulou MD, Georgakilas G, Kostoulas N (2013). DIANA-LncBase: experimentally verified and Computationally predicted microRNA targets on Long non-coding RNAs. Nucleic Acids Res.

[6] Mondal T, Rasmussen M, Pandey GK (2010). Characterization of the RNA content of chromatin. Genome Res.

[7] Wilusz JE, Sunwoo H, Spector DL (2009). Long noncoding RNAs: functional surprises from the RNA world. Genes Dev.

[8] Cesana M, Cacchiarelli D, Legnini I (2011). A long noncoding RNA controls muscle differentiation by functioning as a competing endogenous RNA. Cell.

[9] Karreth FA, Pandolfi PP (2013). ceRNA Cross-Talk in cancer: when ce-bling rivalries go awry. Cancer Discovery.

[10] Taulli R, Loretelli C, Pandolfi PP (2013). From pseudo-ceRNAs to circ-ceRNAs: a tale of cross-talk and competition. Nat Struct Mol Biol.

[11] Salmena L, Poliseno L, Tay Y (2011). A ceRNA hypothesis: the Rosetta Stone of a hidden RNA language?. Cell.

[12] Wu SC, Kallin EM, Zhang Y (2010). Role of H3K27 methylation in the regulation of lncRNA expression. Cell Res.

[13] Yang F, Huo XS, Yuan SX (2013). Repression of the long noncoding RNA-LET by histone deacetylase 3 contributes to hypoxia-mediated metastasis. Mol Cell.

[14] Juan L, Wang G, Radovich M (2013). Potential roles of microRNAs in regulating long intergenic noncoding RNAs. BMC Med Genomics.

[15] Liu Q, Huang J, Zhou N (2013). LncRNA loc285194 is a p53-regulated tumor Suppressor. Nucleic Acids Res.

[16] Wapinski O, Chang HY (2011). Long noncoding RNAs and human disease. Trends Cell Biol.

[17] Raghu G, Collard HR, Egan JJ (2011). An official ATS/ERS/JRS/ALAT statement: idiopathic pulmonary fibrosis: evidence-based guidelines for diagnosis and management. Am J Respir Crit Care Med.

[18] Ding Q, Luckhardt T, Hecker L (2011). New insights into the pathogenesis and treatment of idiopathic pulmonary fibrosis. Drugs.

[19] Cao G, Zhang J, Wang M (2013). Differential expression of long non-coding RNAs in bleomycin-induced lung fibrosis. Int J Mol Med.

[20] Song XD, Liu WL, Xie SY (2013). All-transretinoic acid suppresses epithelial-mesenchymal transition by down-regulating the TGF-β1/Smad3 signaling pathway in bleomycin-induced pulmonary fibrosis in rats. Lab Invest.

[21] Wang M, Zhang J, Song X (2013). Astaxanthin ameliorates lung fibrosis *in vivo* and *in vitro* by preventing transdifferentiation, inhibiting proliferation, and promoting apoptosis of activated cells. Food Chem Toxicol.

[22] Mercer TR, Dinger ME, Mattick JS (2009). Long non-coding RNAs: insights into functions. Nat Rev Genet.

[23] Guttman M, Donaghey J, Carey BW (2011). LincRNAs act in the circuitry controlling pluripotency and differentiation. Nature.

[24] Bertani S, Sauer S, Bolotin E (2011). The noncoding RNA Mistral activates Hoxa6 and Hoxa7 expression and stem cell differentiation by recruiting MLL1 to chromatin. Mol Cell.

[25] He Y, Vogelstein B, Velculescu VE (2008). The antisense transcriptomes of human cells. Science.

[26] Atkinson SR, Marguerat S, Bähler J (2012). Exploring long non-coding RNAs through sequencing. Semin Cell Dev Biol.

[27] Schneider DJ, Wu M, Le TT (2012). Cadherin-11 contributes to pulmonary fibrosis: potential role in TGF-β production and epithelial to mesenchymal transition. FASEB J.

[28] Gutschner T, Diederichs S (2012). The hallmarks of cancer: a long non-coding RNA point of view. RNA Biol.

[29] Schmidt LH, Spieker T, Koschmieder S (2011). The long noncoding MALAT-1 RNA indicates a poor prognosis in non-small cell lung cancer and induces migration and tumor growth. J Thorac Oncol.

[30] Lai MC, Yang Z, Zhou L (2012). Long non-coding RNA MALAT-1 overexpression predicts tumor recurrence of hepatocellular carcinoma after liver transplantation. Med Oncol.

[31] Ying L, Chen Q, Wang Y (2012). Upregulated MALAT-1 contributes to bladder cancer cell migration by inducing epithelial-to-mesenchymal transition. Mol BioSyst.

[32] Sui W, Yan Q, Li H (2013). Genome-wide analysis of long noncoding RNA expression in peripheral blood mononuclear cells of uremia patients. J Nephrol.

[33] Zhu Z, Gao X, He Y (2012). An insertion/deletion polymorphism within RERT-lncRNA modulates hepatocellular carcinoma risk. Cancer Res.

[34] Lomas NJ, Watts KL, Akram KM (2012). Idiopathic pulmonary fibrosis: immunohistochemical analysis provides fresh insights into lung tissue remodelling with implications for novel prognostic markers. Int J Clin Exp Pathol.

[35] Kim KK, Wei Y, Szekeres C (2009). Epithelial cell alpha3beta1 integrin links beta-catenin and Smad signaling to promote myofibroblast formation and pulmonary fibrosis. J Clin Invest.

[36] Ramos C, Becerril C, Montaño M (2010). FGF-1 reverts epithelial-mesenchymal transition induced by TGF-{beta}1 through MAPK/ERK kinase pathway. Am J Physiol Lung Cell Mol Physiol.

[37] Wang Y, Xu Z, Jiang J (2013). Endogenous miRNA sponge lincRNA-RoR regulates Oct4, Nanog, and Sox2 in human embryonic stem cell self-renewal. Dev Cell.

[38] Wang J, Liu X, Wu H (2010). CREB up-regulates long non-coding RNA, HULC expression through interaction with microRNA-372 in liver cancer. Nucleic Acids Res.

[39] Johnsson P, Ackley A, Vidarsdottir L (2013). A pseudogene long-noncoding-RNA network regulates PTEN transcription and translation in human cells. Nat Struct Mol Biol.

[40] Dharap A, Nakka VP, Vemuganti R (2012). Effect of focal ischemia on long noncoding RNAs. Stroke.

[41] Zheng MZ, Qin HD, Yu XJ (2007). Haplotype of gene Nedd4 binding protein 2 associated with sporadic nasopharyngeal carcinoma in the Southern Chinese population. J Transl Med.

[42] Vancheri C (2012). Idiopathic pulmonary fibrosis: an altered fibroblast proliferation linked to cancer biology. Proc Am Thorac Soc.

[43] Cushing L, Kuang PP, Qian J (2011). miR-29 is a major regulator of genes associated with pulmonary fibrosis. Am J Respir Cell Mol Biol.

[44] Kigel B, Rabinowicz N, Varshavsky A (2011). Plexin-A4 promotes tumor progression and tumor angiogenesis by enhancement of VEGF and bFGF signaling. Blood.

[45] Wan YY, Tian GY, Guo HS (2013). Endostatin, an angiogenesis inhibitor, ameliorates bleomycin-induced pulmonary fibrosis in rats. Respir Res.

[46] Pandit KV, Corcoran D, Yousef H (2010). Inhibition and role of let-7d in idiopathic pulmonary fibrosis. Am J Respir Crit Care Med.

