# Supplementary figures and images for: Analysing the relationship between lncRNA and protein-coding gene and the role of lncRNA as ceRNA in pulmonary fibrosis
Source: J Cell Mol Med. 2014 Apr 6;18(6):991–1003. doi: 10.1111/jcmm.12243 (PMC4508140; doi:10.1111/jcmm.12243)

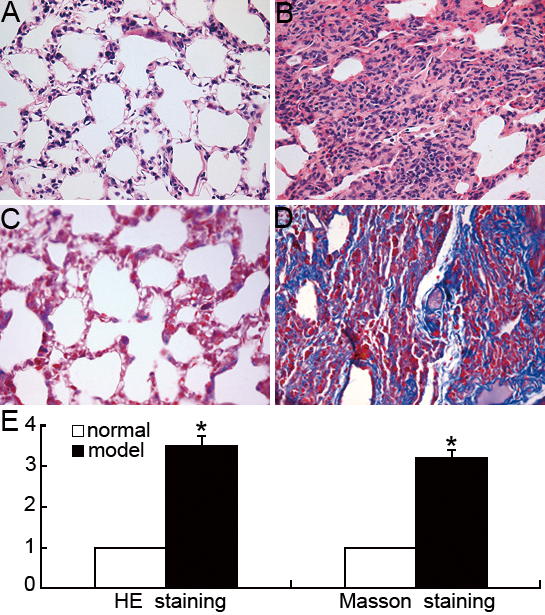

Supplement: Supplementary file 1 [file jcmm0018-0991-sd1.tif]
