# Supplementary material for: Analysing the relationship between lncRNA and protein-coding gene and the role of lncRNA as ceRNA in pulmonary fibrosis
Source: J Cell Mol Med. 2014 Apr 6;18(6):991–1003. doi: 10.1111/jcmm.12243 (PMC4508140; doi:10.1111/jcmm.12243)
Supplement: Supplementary file 2 [file jcmm0018-0991-sd2.doc]

**Supplementary figure legends**

**Supplementary-Table 1.** Differentially expressed lncRNAs screening. To identify differentially expressed lncRNAs, we performed a fold change filtering between the two samples. The threshold is fold change >= 2.0. SeqID: lncRNA name. Fold change: positive value indicates up-regulation and negative value indicates down-regulation. Chr: chromosome No. Which lncRNA is transcribed. Source: the source of LncRNA that is collected from. RefSeq_XR: RefSeq unvalidated non-coding RNA; mouse_ortholog: rat lncRNAs which are obtained by sequence comparison with mouse lncRNAs; UCR: "ultra-conserved region" among human, mouse and rat (http://users.soe.ucsc.edu/~jill/ultra.html); misc_lncRNA: other sources. The list only showed part results of up-regulation and down-regulation expressed lncRNAs in model vs normal.

**Supplementary-Figure 1.** Model identification of pulmonary fibrosis by H&E and Masson staining. (A) H&E staining showed that the alveolar structure was complete and that the alveolar septum was thin in normal lung tissue. (B) H&E staining showed that the alveolar structure was damaged, the pulmonary septa were thickening, and fibroblast focus formation occurred in the model group. (C) Normal lung tissue observed by Masson staining. Collagen was shown as blue in the interstitial lung. (D) Significantly increased collagen fibers were observed by Masson staining in the model group. (E) Grade of pulmonary fibrosis. *Statistically significant difference at P < .05.

| SeqID | Fold  change | chr | strand | source | SeqID | Fold  change | chr | strand | source |
| --- | --- | --- | --- | --- | --- | --- | --- | --- | --- |
| MRAK152421 | 7.63 | 5 | - | mouse_ortholog | XR_005532 | -20.37 | 3 | - | RefSeq_XR |
| AJ006396 | 6.76 | 2 | + | misc_lncRNA | MRAK051854 | -14.47 | 8 | - | mouse_ortholog |
| XR_009519 | 5.66 | 20 | + | RefSeq_XR | MRAK163855 | -8.04 | 5 | + | mouse_ortholog |
| AY973245 | 5.30 | 16 | + | misc_lncRNA | uc.213- | -7.39 | 4 | - | UCR |
| MRAK143591 | 5.09 | 19 | - | mouse_ortholog | MRAK158581 | -6.62 | 14 | + | mouse_ortholog |
| MRAK010032 | 4.84 | 4 | + | mouse_ortholog | MRAK160944 | -6.37 | 5 | + | mouse_ortholog |
| BC089851 | 4.61 | 6 | - | misc_lncRNA | MRAK136348 | -6.26 | 11 | + | mouse_ortholog |
| BC091351 | 4.49 | 6 | + | misc_lncRNA | MRAK018459 | -6.19 | 5 | - | mouse_ortholog |
| MRuc008ljt | 4.43 | 3 | + | mouse_ortholog | BC158675 | -5.90 | 6 | + | misc_lncRNA |

**Supplementary-Table 1** Differentially expressed lncRNAs screening.
